# Chromosome-scale genome assembly of bread wheat’s wild relative *Triticum timopheevii*

**DOI:** 10.1038/s41597-024-03260-w

**Published:** 2024-04-23

**Authors:** Surbhi Grewal, Cai-yun Yang, Duncan Scholefield, Stephen Ashling, Sreya Ghosh, David Swarbreck, Joanna Collins, Eric Yao, Taner Z. Sen, Michael Wilson, Levi Yant, Ian P. King, Julie King

**Affiliations:** 1 Wheat Research Centre, Department of Plant and Crop Sciences, School of Biosciences, University of Nottingham, Loughborough, LE12 5RD UK; 2 Earlham Institute, Norwich Research Park, Norwich, NR4 7UZ UK; 3 Genome Reference Informatics Team, Wellcome Sanger Institute, Wellcome Trust Genome Campus, Hinxton, CB10 1RQ UK; 4 University of California, Department of Bioengineering, Berkeley, CA 94720 USA; 5 United States Department of Agriculture—Agricultural Research Service, Western Regional Research Center, Crop Improvement and Genetics Research Unit, 800 Buchanan St., Albany, CA 94710 USA; 6 University of Nottingham, University Park, Nottingham, NG7 2RD UK

**Keywords:** Comparative genomics, Plant evolution, Genome informatics

## Abstract

Wheat (*Triticum aestivum*) is one of the most important food crops with an urgent need for increase in its production to feed the growing world. *Triticum timopheevii* (2n = 4x = 28) is an allotetraploid wheat wild relative species containing the A^t^ and G genomes that has been exploited in many pre-breeding programmes for wheat improvement. In this study, we report the generation of a chromosome-scale reference genome assembly of *T. timopheevii* accession PI 94760 based on PacBio HiFi reads and chromosome conformation capture (Hi-C). The assembly comprised a total size of 9.35 Gb, featuring a contig N50 of 42.4 Mb and included the mitochondrial and plastid genome sequences. Genome annotation predicted 166,325 gene models including 70,365 genes with high confidence. DNA methylation analysis showed that the G genome had on average more methylated bases than the A^t^ genome. In summary, the *T. timopheevii* genome assembly provides a valuable resource for genome-informed discovery of agronomically important genes for food security.

## Background & Summary

The *Triticum* genus comprises many wild and cultivated wheat species including diploid, tetraploid and hexaploid forms. The polyploid species originated after hybridisation between *Triticum* and the neighbouring *Aegilops* genus (goatgrass). The tetraploid species, *Triticum turgidum* (2n = 4x = 28, AABB), also known as emmer wheat, and *Triticum timopheevii* (2n = 4x = 28, A^t^A^t^GG) are polyphyletic. *Triticum urartu* Thum. ex Gandil (2n = 2x = 14, AA) is the A genome donor for both these species^1^ whereas, the B and G genomes are closely related to the S genome of *Aegilops speltoides*^2^. Both tetraploid species have wild and domesticated forms, i.e., *T. turgidum* L. ssp. *dicoccoides* (Körn. ex Asch. & Graebn.) Thell. and ssp. *dicoccum* (Schrank ex Schübl.) Thell., respectively, and *T. timopheevii* (Zhuk.) Zhuk. ssp. *armeniacum* (Jakubz.) Slageren and ssp. *timopheevii*, respectively. Additionally, tetraploid durum wheat *T. turgidum* L. ssp. *durum* (Desf.) Husn. (2n = 4x = 28, AABB), used for pasta production, and hexaploid bread wheat *Triticum aestivum* L. (2n = 6x = 42, BBAADD) evolved from domesticated emmer wheat with the latter originating through hybridisation with *Aegilops tauschii* (D genome donor) 6,000–7,000 years ago. Hexaploid *Triticum zhukovskyi* (AAGGA^m^A^m^) originated from hybridisation of cultivated *T. timopheevii* and cultivated einkorn *Triticum monococcum*^3^ (2n = 2x = 14, A^m^A^m^).

The G genome is only found in *T. timopheevii* and *T. zhukovskyi* and is virtually identical to the S genome on a molecular level^4,5^ but differs from it, and the B genome, due to a number of chromosomal rearrangements and translocations involving the A^t^ genome^6^. The most studied are the 6A^t^/1 G/4 G and 4 G/4A^t^/3A^t^ translocations in *T. timopheevii*^7–10^.

*Triticum timopheevii* ssp. *timopheevii* has been exploited in various studies for wheat improvement as it has been shown to be an abundant source for genetic variation for many traits such as resistance to leaf rust^11–13^, stem rust^14–16^, powdery mildew^16–18^, Fusarium head blight^19,20^ Hessian fly, Septoria blotch, wheat curl mite and tan spot^21^. It has also been shown to have tolerance to abiotic stresses such as salinity^22,23^ and be a good source for traits affecting grain quality such as milling yield and grain protein^24^ and grain mineral content^25^. During sequence analysis of reference quality assemblies (RQA) of 10 wheat cultivars, recent studies found two of them, cv. LongReach Lancer and cv. Julius, contained major introgressions on Chr2B (among others) potentially originating from *T. timopheevii*^26,27^. Introgressions from *T. timopheevii* have also been found in many other wheat accessions present in genebank collections^28^. Pre-breeding programmes involving the introgression of the whole genome of *T. timopheevii*, in small segments, into bread wheat^10,29^ with diagnostic KASP markers that can track these introgressions in wheat^29,30^ have provided promising new germplasm and tools to the wheat research community.

In this study, we report a chromosome-scale reference genome sequence assembly for *T. timopheevii* by integrating chromatin conformation capture (Hi-C) derived short-reads^31^ with PacBio HiFi long-reads^32^. The assembly was annotated for gene models and repeats. CpG methylation along the chromosomes was inferred from the PacBio CCS data. The high-quality *T. timopheevii* genome assembly obtained in this study provides a reference for the G genome of the *Triticum* genus. This new resource will form the basis to study chromosome rearrangements across different Triticeae species and will be explored to detect A^t^ and G genome introgressions in durum and bread wheat allowing future genome-informed gene discoveries for various agronomic traits.

## Methods

### Plant material, nucleic acid extraction and sequencing

High molecular weight (HMW) DNA was extracted from a young seedling (dark-treated for 48 hours) of *T. timopheevii* accession PI 94760 (United States National Plant Germplasm System, NPGS available at https://npgsweb.ars-grin.gov/gringlobal/search) using a modified Qiagen Genomic DNA extraction protocol (10.17504/protocols.io.bafmibk6)^33^. DNA was sheared to the appropriate size range (15–20 kb) and PacBio HiFi sequencing libraries were constructed by Novogene (UK) Company Limited. Sequencing was performed on 10 SMRT cells of the PacBio Sequel II system in CCS mode with kinetics option to generate ~267 Gb (~28-fold coverage) of long HiFi reads (Supplementary Table S1). Four Hi-C libraries were prepared using leaf samples (from the same plant used for HMW DNA extraction), at Phase Genomics (Seattle, USA) using the Proximo® Hi-C Kit for plant tissues according to the manufacturer’s protocol. The Hi-C libraries were sequenced on an Illumina NovaSeq 6000 S4 platform to generate ~2.8 billion of paired end 150 bp reads (~842 Gb raw data; ~89-fold coverage; Supplementary Table S2).

Total RNA was extracted from seedlings (3-leaf stage), seedlings at dusk, roots, flag leaves, spikes and grains. Flag leaf and whole spike were collected at 7 days post-anthesis and whole grains were collected at 15 days post-anthesis. In brief, 100 mg of ground powder from each tissue was used for RNA isolation using the RNeasy Plant Mini Kit (#74904, QIAGEN Ltd UK). The RNA samples were split into 2 aliquots, one for mRNA sequencing (RNA-Seq) and one for Iso-Seq^34^. Library construction for both types of sequencing was carried out by Novogene (UK) Company Limited. Illumina NovaSeq 6000 S4 platform was used for mRNA sequencing to generate on average 450 million reads (~67 Gb of 2 × 150 bp reads) for each sample (Supplementary Table S3). The second set of RNA aliquots from each of the six tissues were pooled into one sample and sequenced on the PacBio Sequel II system using the Iso-Seq pipeline to generate 4.47 Gb of Iso-Seq data (Supplementary Table S4a) which was analysed using PacBio Iso-Seq analysis pipeline (SMRT Link v12.0.0.177059).

Plants were grown in a glasshouse in 2 L pots containing John Innes No. 2 soil and maintained at 18–25 °C under 16 h light and 8 h dark conditions. All sequencing was carried out by Novogene (UK) Company Limited.

### Cleaning of sequencing data

The HiFi sequencing read files in BAM format were converted and combined into one fastq file using bam2fastq v1.3.1 (available at https://github.com/jts/bam2fastq). Reads with PacBio adapters were removed using cutadapt v4.1^35^ with parameters:–error-rate = 0.1–times = 3–overlap = 35–action = trim–revcomp–discard-trimmed. Hi-C reads were trimmed to remove Illumina adapters using Trimmomatic v0.39^36^ with parameters ILLUMINACLIP:TruSeq 3-PE-2.fa:2:30:10:2:keepBothReads SLIDINGWINDOW:4:20 MINLEN:40 CROP:150.

### Genome size estimation

**Fig. 1:**
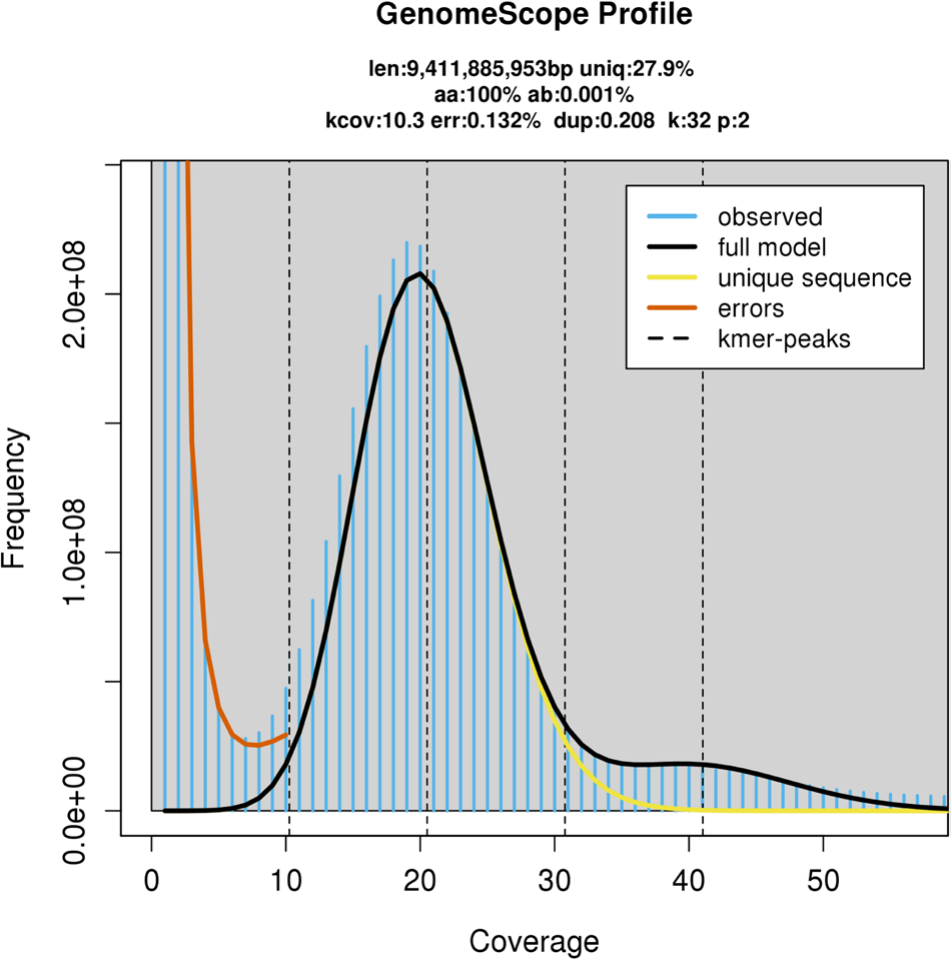
Genomescope profile for 32-mers based on HiFi reads.

The size of the *T. timopheevii* genome was estimated by using k-mer (k = 32) distribution analysis with Jellyfish v2.2.10^37^ on the cleaned HiFi reads^38^. A k-mer count histogram was generated and the size of the *T. timopheevii* genome was estimated as ~9.46 Gb with heterozygosity of 0.001% (Fig. 1), using GenomeScope v2.0^39^ (available at http://qb.cshl.edu/genomescope/genomescope2.0/) with parameters: ploidy = 2, k-mer length = 32, max k-mer coverage = 1000000 and average k-mer coverage = 10.

### Chromosome-scale genome assembly

The cleaned HiFi reads were assembled into the initial set of contigs using hifiasm v.0.16.1^40^ with default parameters and the dataset was assessed using gfastats v1.3.1^41^. The contig assembly had a total size of ~9.41 Gb, with a contig N50 value of 43.12 Mb. Genome completeness was assessed for the contig assembly using the Benchmarking Universal Single-Copy Orthologs (BUSCO v5.3.2)^42^ program with the embryophyta_odb10 database which yielded 99% of the complete BUSCO genes. Contaminants (contigs other than those categorised as Streptophyta or no hit) were identified using BlobTools v1.1.1^43^ and removed.

To achieve chromosome-level assembly, the trimmed Hi-C data^44^ was mapped onto the decontaminated contig assembly using the Arima Genomics® mapping pipeline (available at https://github.com/ArimaGenomics/mapping_pipeline) and chromosome construction was conducted using the Salsa2^45^ pipeline (available at https://github.com/marbl/SALSA) with default parameters and GATC as the cutting site for the restriction enzyme (DpnII). The Hi-C contact map for the scaffold assembly was constructed using PretextMap v0.1.9 and the chromatin contact matrix was manually corrected using PretextView v0.2.5 by following the Rapid Curation pipeline^46^ (https://gitlab.com/wtsi-grit/rapid-curation). The curated assembly was assessed using gfastats to consist of 14 pseudomolecules and 1656 unplaced scaffolds with a total length of 9,350,839,849 bp (including gaps) and a contig N50 of 42.4 Mb (Table 1). The orientation and the chromosome name of each pseudomolecule were determined based on homology with the wheat cv. Chinese Spring assembly RefSeq 2.1^47^ A and B subgenomes, using dotplot comparison of sequence alignments produced by MUMmer’s (v3.23^48^) nucmer aligner and viewed on Dot (available at https://github.com/marianattestad/dot). The pseudomolecules were thus, renamed into the 14 *T. timopheevii* chromosomes, seven A^t^ genome chromosomes with a total length of ~4.85 Gb and consisting of 119 contigs and seven G genome chromosomes with a total length of ~4.40 Gb and consisting of 529 contigs (Table 2).

**Table 1:** Summary statistics for genome assembly of *Triticum timopheevii*.

Assembly characteristics	Value
Number of scaffolds	1,670
Total scaffold length (bp)	9,350,839,849
Scaffold N50 (bp)	671,191,297
Largest scaffold (bp)	771,176,557
No. of contigs	2,304
Total contig length (bp)	9,350,587,949
Average contig length (bp)	4,058,415
Contig N50 (bp)	42,410,373
Largest contig (bp)	311,469,246
GC content (%)	46
BUSCO evaluation (% of complete BUSCO genes)	99.1

**Table 2:** Statistics of the *Triticum timopheevii* pseudomolecules.

Chromosome	Length (bp)	Number of contigs	Number of gene models
Chr1A^t^	614,431,332	14	9,982
Chr1G	495,016,746	50	8,777
Chr2A^t^	767,071,137	10	12,729
Chr2G	671,256,291	72	13,941
Chr3A^t^	670,741,101	10	9,489
Chr3G	671,191,297	75	13,452
Chr4A^t^	771,176,557	23	12,878
Chr4G	643,128,204	68	9,936
Chr5A^t^	694,350,238	12	11,821
Chr5G	641,290,954	78	13,079
Chr6A^t^	585,824,631	33	9,011
Chr6G	589,079,669	87	11,406
Chr7A^t^	745,638,687	17	12,863
Chr7G	692,654,486	99	14,851
Unplaced scaffolds	97,988,519	1656	2,110
**Total**	**9,350,839,849**	**2,304**	**166,325**

### Organellar genome assembly

*De novo* assembly of the organelle genomes was carried out using the Oatk pipeline (available at https://github.com/c-zhou/oatk) with the cleaned HiFi reads. The circular chloroplast and mitochondrial contigs were assembled with a total size of 136,158 bp and 443,464 bp, respectively. Any unanchored contigs that aligned to these extranuclear genomes were removed from the final assembly.

### Genome annotation

Gene models were generated from the *T. timopheevii* assembly using REAT - Robust and Extendable eukaryotic Annotation Toolkit (https://github.com/EI- CoreBioinformatics/reat) and Minos (https://github.com/EI-CoreBioinformatics/minos) which make use of Mikado^49^ (https://github.com/EI-CoreBioinformatics/mikado), Portcullis (https://github.com/EI-CoreBioinformatics/portcullis) and many third-party tools (listed in the above repositories). A consistent gene naming standard^50^ was used to make the gene models uniquely identifiable.

1. **Repeat identification**

Repeat annotation was performed using EI-Repeat version 1.3.4 pipeline (https://github.com/EI- CoreBioinformatics/eirepeat) which uses third party tools for repeat calling. In the pipeline, RepeatModeler (v1.0.11 - http://www.repeatmasker.org/RepeatModeler/) was used for *de novo* identification of repetitive elements from the assembled *T. timopheevii* genome. High copy protein coding genes potentially included in the RepeatModeler library were identified and effectively removed by running RepeatMasker v4.0.7 using a curated set of high confidence *T. aestivum* coding genes to hard mask the RepeatModeler library; transposable element genes were first excluded from the *T. aestivum* coding gene set by running TransposonPSI (r08222010). Unclassified repeats were searched in a custom BLAST database of organellar genomes (mitochondrial and chloroplast sequences from *Triticum* in the NCBI nucleotide division). Any repeat families matching organellar DNA were also hard-masked. Repeat identification was completed by running RepeatMasker v4.0.72 with a RepBase embryophyte library and with the customized RepeatModeler library (i.e. after masking out protein coding genes), both using the -nolow setting. Overall, 78.43% of the assembly was classified as repetitive sequences (Table 3). The consolidated set of repeat features (i.e. RepeatMasker outputs from the embryophyte and customized RepeatModeler libraries) were given as input to the evidence guided gene prediction (REAT prediction) and gene model consolidation (Minos) steps. All other annotation steps utilised the unmasked genome.

**Table 3:** Classification of repeat annotation in *Triticum timopheevii*.

	Class	Number of elements	Length occupied (bp)	Percentage of sequence
Retrotransposons	SINEs	20,589	1,759,774	0.02
	LINEs	150,497	116,697,520	1.25
	LTRs: Copia	535,455	1,620,870,187	17.33
	LTRs: Gypsy	1,690,034	3,873,777,180	41.43
	LTRs: Unknown	1,501,064	139,512,022	1.49
DNA transposons	hobo-Activator	20,177	6,117,182	0.07
	Tc1-IS630-Pogo	118,082	16,100,160	0.17
	Tourist/Harbinger	54,914	16,353,000	0.17
	Other	1,582,537	948,759,196	10.15
Unclassified	—	1,164,179	593,808,513	6.35
**Total**		**6,837,528**	**7,333,754,734**	**78.43**

2. **Reference guided transcriptome reconstruction**
  Gene models were derived from the RNA-Seq reads, Iso-Seq transcripts (122,253 high quality and 82 low quality isoforms; Supplementary Table S4b) and Full-Length Non- Concatamer Reads (FLNC) using the REAT transcriptome workflow. HISAT2 v2.2.1^51^ was selected as the short read aligner with Iso-Seq transcripts aligned with minimap2 v2.18-r1015^52^, maximum intron length was set as 50,000 bp and minimum intron length to 20 bp. Iso-Seq alignments were required to meet 95% coverage and 90% identity. High-confidence splice junctions were identified by Portcullis v 1.2.4^53^. RNA-Seq Illumina reads were assembled for each tissue with StringTie2 v2.1.5^54^ and Scallop v0.10.5^55^, while FLNC reads were assembled using StringTie2 (Supplementary Table S5). Gene models were derived from the RNA-Seq assemblies and Iso-Seq and FLNC alignments with Mikado. Mikado was run with all Scallop, StringTie2, Iso-Seq and FLNC alignments and a second run with only Iso-Seq and FLNC alignments (Supplementary Table S6).
3. **Cross-species protein alignment**
  Protein sequences from 10 Poaceae species (Supplementary Table S7) were aligned to the *T. timopheevii* assembly using the REAT Homology workflow with options–annotation_filters aa_len–alignment_species Angiosp–filter_max_intron 20000–filter_min_exon 10–alignment_filters aa_len internal_stop intron_len exon_len splicing–alignment_min_coverage 90–junction_f1_filter 40–post_alignment_clip clip_term_intron-exon–term5i_len 5000–term3i_len 5000–term5c_len 36–term3c_len 36. The REAT Homology workflow aligns proteins with spaln v2.4.7^56^ and filters and generates metrics to remove misaligned proteins. Simultaneously, the same protein set were also aligned using miniprot v0.3^57^ and similarly filtered as in the REAT homology workflow. The aligned proteins from both methods were clustered into loci and a consolidated set of gene models were derived via Mikado.
4. **Evidence guided gene prediction**
  The evidence guided annotation of protein coding genes based on repeats, RNA-Seq mappings, transcript assembly and alignment of protein sequences was created using the REAT prediction workflow. The pipeline has four main steps: (1) the REAT transcriptome and homology Mikado models are categorised based on alignments to UniProt proteins to identify models with likely full-length CDS and which meet basic structural checks i.e., having complete but not excessively long UTRs and not exceeding a minimum CDS/cDNA ratio. A subset of gene models is then selected from the classified models and used to train the AUGUSTUS gene predictor^58^; (2) Augustus is run in both *ab initio* mode and with extrinsic evidence generated in the REAT transcriptome and homology runs (repeats, protein alignments, RNA-Seq alignments, splice junctions, categorised Mikado models). Three evidence guided AUGUSTUS predictions are created using alternative bonus scores and priority based on evidence type; (3) AUGUSTUS models, REAT transcriptome/homology models, protein and transcriptome alignments are provided to EVidenceModeler^59^ (EVM) to generate consensus gene structures; (4) EVM models are processed through Mikado to add UTR features and splice variants.
5. **Projection of gene models from Triticum aestivum**
  A reference set of hexaploid wheat gene models was derived from public gene sets (IWGSC^60^ and 10+ wheat^26^) projected onto the IWGSC RefSeq v1.0 assembly^60^; a filtered and consolidated set of models was derived with Minos, with a primary model defined for each gene. Models were scored on a combination of intrinsic gene structure characteristics, evidence support (protein and transcriptome data) and consistency in gene structure across the input gene models. The Minos primary models were classified as full-length or partial based on alignment to a filtered magnoliopsida Swiss-Prot TrEMBL database. This assignment, together with criteria for gene structure characteristics and the original confidence classification, was used to classify models into 6 categories (Platinum, Gold, Silver, Bronze, Stone and Paper), with Platinum being the highest confidence category for models assessed as full-length, with an original confidence classification of “high”, meeting structural checks for number of UTR and CDS/cDNA ratio and which were assessed as consistently annotated across the input gene sets. Reclassification resulted in 55,319 Platinum, 24,789 Gold, 11,968 Silver, 61,845 Bronze, 110,518 Stone and 115,336 Paper genes. The four highest confidence categories Platinum, Gold, Silver and Bronze were projected onto the *T. timopheevii* assembly with Liftoff v1.5.1^61^, only those models transferred fully with no loss of bases and identical exon/intron structure were retained (https://github.com/lucventurini/ei-liftover). Similarly, high confidence genes annotated in the hexaploid wheat cv. Chinese Spring RefSeq v2.1 assembly^47^ were projected onto the *T. timopheevii* genome assembly with Liftoff, and only those models transferred fully with no loss of bases and identical exon/intron structure were retained. Among these, gene models with the attribute “manually_curated” in the original Refseq v2.1 assembly were extracted as a set.
6. **Gene model consolidation**

The final set of gene models was selected using Minos (Table 4). Minos is a pipeline that generates and utilises metrics derived from protein, transcript, and expression data sets to create a consolidated set of gene models. In this annotation, the following gene models were filtered and consolidated into a single set of gene models using Minos:

1. The three alternative evidence guided Augustus gene builds described earlier.
2. The gene models derived from the REAT transcriptome runs described earlier.
3. The gene models derived from the REAT homology runs described earlier.
4. The gene models derived from the REAT prediction run (AUGUSTUS and EVM-Mikado) described earlier.
5. The gene models derived from projecting public and curated *T. aestivum* gene models of varying confidence levels onto the *T. timopheevii* genome as described earlier.
6. IWGSC Refseq v2.1 models identified as “manually_curated” projected onto the *T. timopheevii* genome as described earlier.

**Table 4:** Summary statistics for the final structural annotation of the *T. timopheevii* genome.

Stat	Value
Number of genes	166,325
Number of Transcripts	218,100
Transcripts per gene	1.31
Number of monoexonic genes	51,702
Monoexonic transcripts	53,192
Transcript mean size cDNA (bp)	1,658.27
Transcript median size cDNA (bp)	1412
Min cDNA	96
Max cDNA	20,589
Total exons	997,779
Exons per transcript	4.57
Exon mean size (bp)	362.47
CDS mean size (bp)	277.55
Transcript mean size CDS (bp)	1,171.61
Transcript median size CDS (bp)	957
Min CDS	0
Max CDS	20,283
Intron mean size (bp)	628.4
5’UTR mean size (bp)	182.93
3’UTR mean size (bp)	294.58

Gene models were classified as biotypes protein_coding_gene, predicted_gene and transposable_element_gene, and assigned as high or low confidence (Table 5) based on the criteria below:

- **High confidence (HC) protein_coding_gene:** Any protein coding gene where any of its associated gene models have a BUSCO v5.4.7^62^ protein status of Complete/Duplicated OR have diamond v0.9.36 coverage (average across query and target coverage) >= 90% against the listed Poaceae protein datasets (Supplementary Table S7) or UniProt magnoliopsida proteins. Or alternatively have average blastp coverage (across query and target coverage) >= 80% against the listed protein datasets/UniProt magnoliopsida AND have transcript alignment F1 score (average across nucleotide, exon and junction F1 scores based on RNA-Seq transcript assemblies) >= 60%.
- **Low confidence (LC) protein_coding_gene:** Any protein coding gene where all its associated transcript models do not meet the criteria to be considered as high confidence protein coding transcripts.
- **HC transposable_element_gene:** Any protein coding gene where any of its associated gene models have coverage >= 40% against the combined interspersed repeats (see section 1).
- **LC transposable_element_gene:** Any protein coding gene where all its associated transcript models do not meet the criteria to be considered as high confidence and assigned as a transposable_element_gene (see c).
- **LC predicted_gene:** Any protein coding gene where all the associated transcript models do not meet the criteria to be considered as high confidence protein coding transcripts. In addition, where any of the associated gene models have average blastp coverage (across query and target coverage) <30% against the listed protein datasets AND having a protein-coding potential score <0.25 calculated using CPC2 0.1^63^.
- **LC ncRNA gene:** Any gene model with no CDS features AND a protein-coding potential score <0.3 calculated using CPC2 0.1.
- **Discarded models:** Any models having no BUSCO protein hit AND a protein alignment score (average across nucleotide, exon and junction F1 scores based on protein alignments) <0.2 AND a transcript alignment F1 score (average across nucleotide, exon and junction F1 scores based on RNA-Seq transcript assemblies) <0.2 AND a diamond coverage (target coverage) <0.3 AND Kallisto v0.44^64^ expression score <0.2 from across RNA-Seq reads OR having short CDS <30 bps. Any ncRNA genes (no CDS features) not meeting the ncRNA gene requirements (f) were also excluded.

**Table 5:** Minos classified gene models.

Biotype	Confidence	Gene	Transcript
protein_coding_gene	Low	73,844	79,329
protein_coding_gene	High	67,107	112,338
transposable_element_gene	Low	15,871	16,231
predicted_gene	Low	4,974	5,033
transposable_element_gene	High	3,258	3,410
ncRNA_gene	Low	1,271	1,759
**Total**		**166,325**	**218,100**

**Fig. 2:**
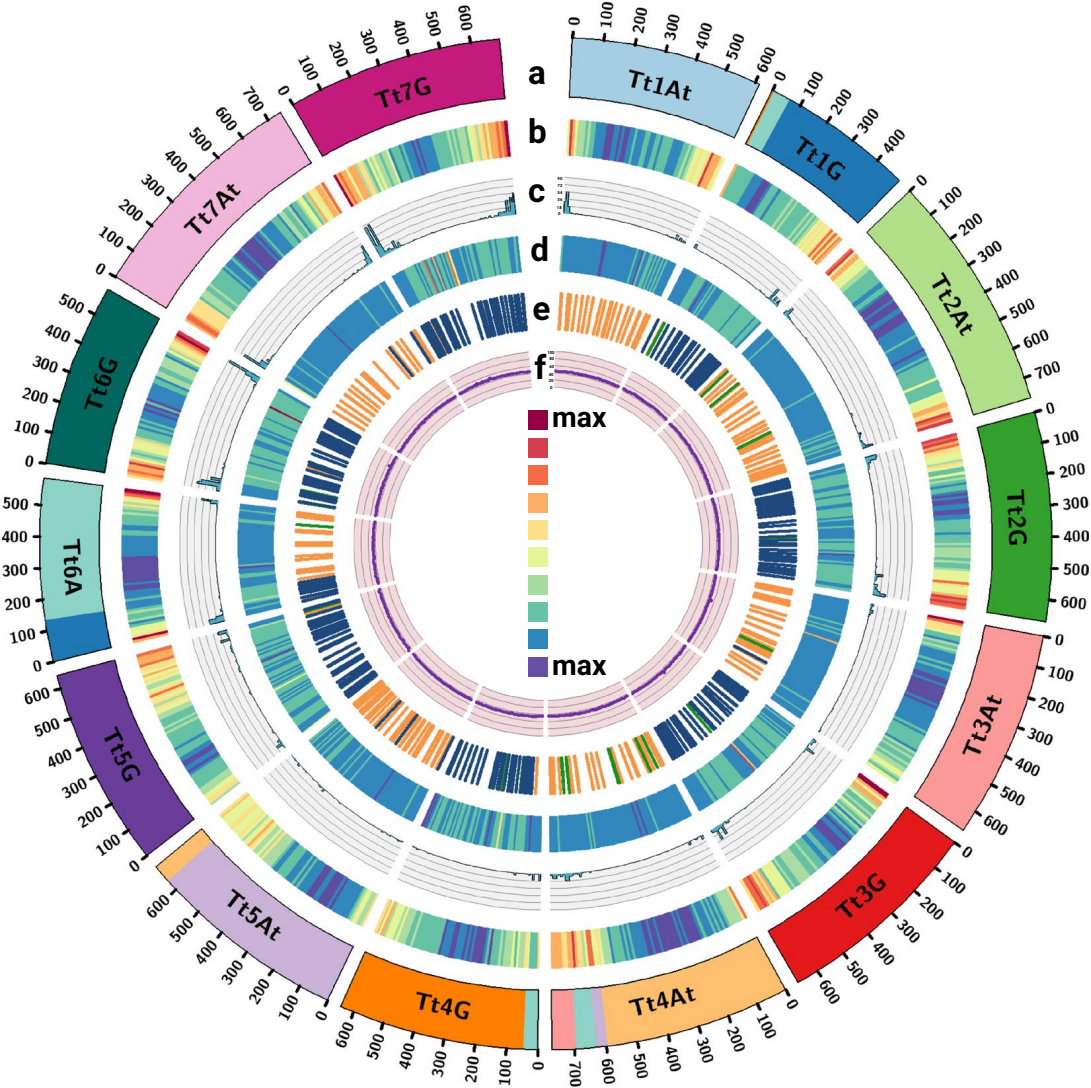
Circos plot^84^ of features of the chromosome-scale assembly of *T. timopheevii* showing (**a**) major translocations with the T. timopheevii genome as observed through collinearity analysis against *T. turgidum*, (**b**) gene density (of all gene models), (**c**) NLR density (max count 87), (**d**) DNA methylation (5mC modification) density, (**e**) distribution of KASP markers based on SNPs with bread wheat cv. Chinese Spring^29^ and (**f**) GC content (in %). Tt in chromosome name represents *T. timopheevii*. Y-axis for tracks c and f have an interval of 18 and 20 units, respectively.

Gene model distribution across the pseudomolecules and unplaced scaffolds is shown in Table 2 and gene density of 164,617 protein coding genes across the *T. timopheevii* genome was calculated using deepStats v0.4^65^ in 10 Mb bins (Fig. 2b).

7. **Functional annotation**

All proteins were annotated using AHRD v.3.3.3 (available at https://github.com/groupschoof/AHRD/blob/master/README.textile). Sequences were compared against the reference proteins (Arabidopsis thaliana TAIR10, TAIR10_pep_20101214_updated.fasta.gz - https://www.araport.org) and the UniProt viridiplantae sequences^66^ (data download 06-May-2023), both Swiss-Prot and TrEMBL datasets using blastp v2.6.0 with an e-value of 1e- 5. InterproScan v5.22.61^67^ results were also provided to AHRD. The standard AHRD example configuration file path test/resources/ahrd_example_input_go_prediction.yml, distributed with the AHRD tool, was adapted apart from the location of input and output files. The GOA mapping from UniProt (ftp://ftp.ebi.ac.uk/pub/databases/GO/goa/UNIPROT/goa_uniprot_all.gaf.gz) was included as parameter ‘gene_ontology_result’. The interpro database (ftp://ftp.ebi.ac.uk/pub/databases/interpro/61.0/interpro.xml.gz) was included as parameter ‘interpro_database’. The parameter ‘prefer_reference_with_go_annos’ was changed to ‘false’ and the blast database specific weights used were:

blast_dbs:

swissprot:

weight: 100

description_score_bit_score_weight: 0.2

trembl:

weight: 50

description_score_bit_score_weight: 0.4

tair:

weight: 50

description_score_bit_score_weight: 0.4

Since *T. timopheevii* is known as an important source for genetic variation for resistance against major diseases of wheat as described above and as the majority of cloned disease-resistance genes encode nucleotide-binding leucine-rich repeats (NLRs)^68,69^, we validated the annotation of all gene models annotated as NB-ARC domain-containing/disease resistance proteins in the genome assembly (2399 gene models) using NLR-Annotator v2^70^ and found an additional 166 NLRs (total 2565). We plotted the genomic distribution of the larger set (Fig. 2c), by calculating the density in 10 Mb bins using deepStats v0.4, which shows concentration of these NLRs at mostly distal ends of the chromosomes of *Triticum timopheevii*.

### Generation of PacBio DNA methylation profile

Methylation in CpG context was inferred with ccsmeth v0.3.2^71^, using the kinetics data from PacBio CCS subreads obtained during HMW DNA sequencing. The methylation prediction for CCS reads were called using the model “model_ccsmeth_5mCpG_call_mods_attbigru2s_b21.v2.ckpt”. The reads with the MM + ML tags were aligned to the pseudomolecules in the *T. timopheevii* assembly using BWA v0.7.17^72^. The methylation frequency was calculated at genome level with the modbam files and the aggregate mode of ccsmeth with the model “model_ccsmeth_5mCpG_aggregate_attbigru_b11.v2p.ckpt”. The genomic distribution of 5mC modifications across *T. timopheevii* (Fig. 2d) shows that G genome chromosomes have more methylation with an average of ~401.8 Kbp methylated bases per 10 Mb bin as compared to the A^t^ genome chromosomes with an average of ~385.5 Kbp per 10 Mb bin (calculated using deepStats v0.4).

## Data Records

The raw sequence files for the HiFi, Hi-C, RNA-Seq and IsoSeq reads were deposited in the European Nucleotide Archive (ENA) under accession number PRJEB71660^73^. The final chromosome-scale assembly consisting of the nuclear and organelle genomes was deposited at ENA under the accession number GCA_963921465.1^74^.

The genome assemblies, gene model and repeat annotations, methylation profile and Hi-C contact map are also available at on DRYAD Digital Repository^75^.

## Technical Validation

### Assessment of genome assembly and annotation

**Fig. 3:**
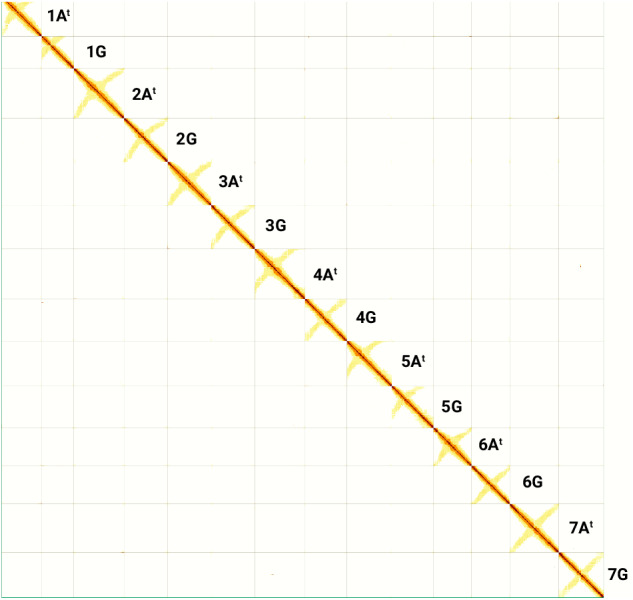
Contact map after the integration of the Hi-C data and manual correction using PretextView.

The final curated assembly was assessed by mapping the trimmed Hi-C reads to the post-curated assembly (as described above for scaffolding) and generating a final Hi-C contact map using PretextMap v0.1.9 and viewed using PretextView v0.2.5. It showed a dense dark blue pattern along the diagonal revealing no potential mis-assemblies (Fig. 3). The anti-diagonals in the Hi-C contact matrix were expected and have been reported for other relatively large plant genomes such as those from the Triticeae tribe^76,77^ as they correspond to the typical Rabl configuration of Triticeae chromosomes^78,79^.

The BUSCO v5.3.2^42^ (-l poales_odb10) score of 99.1% (0.1% fragmented and 0.8% missing BUSCOs; Supplementary Table S8a) at the genome level indicates a high completeness of the *T. timopheevii* assembly. The quality of the *T. timopheevii* assembly was assessed with Merqury^80^ based on the PacBio HiFi reads using 31-mers. The QV (consensus quality value) and k-mer completeness scores were 65.5 and 97.8%, respectively. The quality of the assembly was further assessed by determining the LTR Assembly Index (LAI) and attainment of a value of 13.62 suggests that the assembly meets the criteria for a reference quality genome^81^ indicating a high level of accuracy and completeness in capturing genomic features, particularly those related to LTR retrotransposons.

Completeness of the gene model prediction was also evaluated using BUSCO (-l poales_odb10) and produced a score of 99.9% (0.0% fragmented and 0.1% missing BUSCOs; Supplementary Table S8b). The number of HC gene models (70,365) is in the range of a tetraploid Triticeae species (34,000–43,000 high-confidence gene models per haploid genome)^82^.

Of the total 14 chromosomes, we found telomeric repeats on both ends for 5 chromosomes (1A^t^, 2 G, 3A^t^, 6A^t^, and 7 G) and on one end for 7 chromosomes (1GL, 2A^t^S, 3GL, 4GS, 5GL, 6GL and 7A^t^L).

## Usage Notes

A genome browser for the assembly of *T. timopheevii* generated in this study is currently being hosted at GrainGenes^83^ (https://wheat.pw.usda.gov/jb?data=/ggds/whe-timopheevii) with tracks for annotated gene models and repeats and BLAST functionality available at https://wheat.pw.usda.gov/blast/.

### Supplementary information

Supplementary Information

## Data Availability

All software and pipelines were executed according to the manual and protocol of published tools. No custom code was generated for these analyses.
