# Supplementary material for: Chromosome-scale genome assembly of bread wheat’s wild relative Triticum timopheevii
Source: Sci Data. 2024 Apr 23;11:420. doi: 10.1038/s41597-024-03260-w (PMC11039740; doi:10.1038/s41597-024-03260-w)
Supplement: Supplementary file 1 — Supplementary Information [file 41597_2024_3260_MOESM1_ESM.pdf]

Supplementary Information

Chromosome-scale genome assembly of bread wheat's wild relative *Triticum timopheevii*

Surbhi Grewal[1], Cai-yun Yang[1], Duncan Scholefield[1], Stephen Ashling[1], Sreya Ghosh[2], David Swarbreck[2], Joanna Collins[3], Eric Yao[4,5], Taner Z. Sen[4,5], Michael Wilson[6], Levi Yant[6], Ian P. King[1] and Julie King[1]

1. Wheat Research Centre, Department of Plant and Crop Sciences, School of Biosciences, University of Nottingham, Loughborough, LE12 5RD, UK
2. Earlham Institute, Norwich Research Park, Norwich NR4 7UZ, UK
3. Genome Reference Informatics Team, Wellcome Sanger Institute, Wellcome Trust Genome Campus, Hinxton, CB10 1RQ, UK
4. University of California, Department of Bioengineering, Berkeley, CA, 94720, USA
5. United States Department of Agriculture—Agricultural Research Service, Western Regional Research Center, Crop Improvement and Genetics Research Unit, 800 Buchanan St., Albany, CA 94710, USA
6. University of Nottingham, University Park, Nottingham, NG7 2RD

Corresponding author: Surbhi Grewal (surbhi.grewal@nottingham.ac.uk)

Table of Contents

Table S1. Statistics of HiFi reads. ***2***

Table S2. Statistics of HiC reads. ***3***

Table S3. Statistics of mRNA sequencing. ***4***

Table S4. Statistics of Iso-Seq sequencing (a) and initial analysis (b) using the PacBio Iso-Seq pipeline. ***5***

Table S5. Reference guided transcriptome assembly statistics for short read transcriptome data assembled with Stringtie and Scallop for FLNC reads assembled with StringTie. ***6***

Table S6. REAT Transcriptome Mikado consolidated gene sets, gene model statistics. ***7***

Table S7. List of Species used for cross species protein alignment ***8***

Table S8. BUSCO evaluation results of (a) genome assembly and (b) gene model prediction ***9***

Table S1. Statistics of HiFi reads.

Sample	Cell	HiFi reads bases (bp)	Total bases (Gb)	HiFi reads number	Average HiFi reads length	N50
S95	S95_m64165_220422_130103	26,589,037,091	199.12	1,842,033	14,434	16,850
S95	S95_m64165_220425_132611	10,060,161,095		640,383	15,709	16,775
S95	S95_m64164_220605_052635	22,093,742,922		1,684,360	13,116	15,031
S95	S95_m64164_220610_124716	21,036,575,173		1,478,325	14,230	15,571
S95	S95_m64164_220611_190014	28,784,821,607		1,886,131	15,261	15,752
S95	S95_m64164_220613_011317	32,085,349,484		2,123,969	15,106	15,590
S95	S95_m64164_220615_140016	27,316,613,580		1,919,234	14,233	15,115
S95	S95_m64165_220615_155227	31,158,294,078		2,066,458	15,078	15,482
P95	P95_m64165_221012_052633	35,716,109,179	67.53	1,962,144	18,202	18,092
P95	P95_m64267e_221023_073301	31,810,292,249		1,782,735	17,843	17,713
	**Total**	266,650,996,458	267	17,385,772		

Table S2. Statistics of HiC reads.

Sample	Library_Flowcell_Lane	Raw reads (bp)	Raw data (Gb)	Effective(%)	Error(%)	Q20(%)	Q30(%)	GC(%)
sp22161_1	EKDL220006552-1a_HKG5JDSX3_L4	177,231,714	214.1	100	0.03	96.32	90.45	45.66
sp22161_1	EKDL220006552-1a_HKHCTDSX3_L1	1,250,355,254		99.35	0.03	94.71	88.33	46.04
sp22161_2	EKDL220006549-1a_HKHCTDSX3_L1	1,350,683,438	202.6	99.35	0.03	94.95	88.94	46.69
sp22161_3	EKDL220006550-1a_HKG5JDSX3_L4	137,595,592	212.1	99.99	0.03	96.87	92.09	46.45
sp22161_3	EKDL220006550-1a_HKHCTDSX3_L1	1,276,257,308		99.32	0.03	95.06	89.42	46.62
sp22161_4	EKDL220006551-1a_HKHCTDSX3_L1	1,144,706,830	213.4	99.37	0.03	94.37	87.99	46.5
sp22161_4	EKDL220006551-1a_HKG2JDSX3_L3	277,860,482		100	0.03	95.93	89.76	46.15
	**Total**	5,614,690,618	842.2					

Table S3. Statistics of mRNA sequencing.

Sample*	Library_Flowcell_Lane	Raw reads (bp)	Raw data (Gb)	Effective(%)	Error(%)	Q20(%)	Q30(%)	GC(%)
Tim_Grn	EKRN230001853-1A_HMTFTDSX5_L2	29,753,674	61.5	96.63	0.03	96.03	90.63	52.03
Tim_Grn	EKRN230001853-1A_HMTHFDSX5_L2	380,224,686		97.55	0.03	97.17	93.17	52.72
Tim_RT	EKRN230001854-1A_HMTHFDSX5_L2	528,260,132	79.2	98.38	0.02	98.08	94.75	55.62
Tim_Ypl_am	EKRN230001855-1A_HMTHFDSX5_L2	489,608,798	73.4	98.56	0.02	98.12	94.84	56.85
Tim_Ypl_pm	EKRN230001856-1A_HMTHFDSX5_L2	352,306,000	62.5	98.78	0.03	97.78	93.98	56.07
Tim_Ypl_pm	EKRN230001856-1A_HMTFTDSX5_L2	64,037,984		98.27	0.03	96.46	90.91	55.23
Tim_Spk	EKRN230001857-1A_HMTHFDSX5_L2	446,443,460	67	98.57	0.02	98.03	94.6	55.08
Tim_Flg	EKRN230001852-1A_HMVJWDSX5_L2	406,828,476	61	97.87	0.03	97.37	93.15	56.65
	**Total**	2,697,463,210	405					

*Sample_Code	Sample_Description
Tim_Flg	Timopheevii Flag Leaf
Tim_Grn	Timopheevii Grains
Tim_RT	Timopheevii Roots
Tim_Ypl_am	Timopheevii seedlings dawn
Tim_Ypl_pm	Timopheevii seedlings dusk
Tim_Spk	Timopheevii Spike

Table S4. Statistics of Iso-Seq sequencing (a) and initial analysis (b) using the PacBio Iso-Seq pipeline.

(a)

Sample	Cell	HiFi reads bases (bp)	Total bases(Gb)	HiFi reads number	Average HiFi reads length	N50
Tim_pool	Tim_pool_m64165_230216_134625	4,468,440,060	4.47	2,179,791	2,049	2,216

(b)

CCS Analysis Read Classification	Values
Reads	2,179,791
Reads with 5' and 3' Primers	2,013,464
Non-Concatamer Reads with 5' and 3' Primers	2,010,455
Non-Concatamer Reads with 5' and 3' Primers and Poly-A Tail	2,008,983
Mean Length of Full-Length Non-Concatamer Reads	1,974
Unique Primers	1
Mean Reads per Primer	2,013,464
Max. Reads per Primer	2,013,464
Min. Reads per Primer	2,013,464
Reads without Primers	166,327
Percent Bases in Reads with Primers	0.9243
Percent Reads with Primers	0.9237
Number of High-Quality Isoforms	122,253
Number of Low-Quality Isoforms	82

Table S5. Reference guided transcriptome assembly statistics for short read transcriptome data assembled with Stringtie and Scallop for FLNC reads assembled with StringTie.

Stat	Tim_Flg (StringTie)	Tim_Grn (StringTie)	Tim_RT (StringTie)	Tim_Spk (StringTie)	Tim_Ypl_am (StringTie)	Tim_Ypl_pm (StringTie)	Tim_Flg (Scallop)	Tim_Grn (Scallop)	Tim_RT (Scallop)	Tim_Spk (Scallop)	Tim_Ypl_am (Scallop)	Tim_Ypl_pm (Scallop)	FLNC (StringTie)
**Number of genes**	64,440	74,303	74,018	80,533	78,710	74,985	88,176	96,768	110,719	120,211	119,355	108,452	38,266
**Number of Transcripts**	107,001	116,848	122,246	131,678	121,371	119,890	171,587	178,280	207,036	219,391	199,944	194,294	47,645
**Transcripts per gene**	1.66	1.57	1.65	1.64	1.54	1.6	1.95	1.84	1.87	1.83	1.68	1.79	1.25
**Number of monoexonic genes**	9,042	8,029	11,696	13,082	16,772	12,699	9,722	9,324	16,010	18,183	28,058	16,834	2,267
**Monoexonic transcripts**	9,608	8,429	12,399	13,995	17,672	13,479	13,582	12,364	22,207	24,503	35,648	22,086	2,613
**Transcript mean size cDNA (bp)**	1,870.56	1,695.45	1,804.84	1,692.87	1,565.93	1,687.33	1,701.11	1,611.81	1,642.09	1,500.57	1,343.54	1,481.63	2,288.66
**Transcript median size cDNA (bp)**	1,589	1,458	1,555	1,458	1,370	1,458	1,412	1,348	1,364	1,243	1,104	1,230	2,083
**Min cDNA**	200	200	200	200	200	200	200	205	200	200	200	200	214
**Max cDNA**	29,570	24,804	22,841	22,800	18,140	24,471	27,601	24,173	19,679	22,899	19,721	24,230	11,557
**Total exons**	590,973	633,237	673,868	691,273	612,859	629,905	859,672	896,823	1,000,394	1,023,926	858,851	903,298	314,594
**Exons per transcript**	5.52	5.42	5.51	5.25	5.05	5.25	5.01	5.03	4.83	4.67	4.3	4.65	6.6
**Exon mean size (bp)**	338.68	312.85	327.42	322.47	310.12	321.15	339.53	320.41	339.84	321.52	312.78	318.69	346.62
**Intron mean size (bp)**	627.99	636.98	637.24	628.67	631.46	642.5	632.04	655.41	625.35	623.85	641.96	634.87	499.34

Sample_Code	Sample_Description
Tim_Flg	Timopheevii Flag Leaf
Tim_Grn	Timopheevii Grains
Tim_RT	Timopheevii Roots am
Tim_Ypl_am	Timopheevii seedlings dawn
Tim_Ypl_pm	Timopheevii seedlings dusk
Tim_Spk	Timopheevii Spike

Table S6. REAT Transcriptome Mikado consolidated gene sets, gene model statistics.

Stat	mikado_long.loci	mikado_all.loci
**Number of genes**	38,734	76,872
**Number of Transcripts**	43,617	118,829
**Transcripts per gene**	1.13	1.55
**Number of monoexonic genes**	4,965	11,082
**Monoexonic transcripts**	5,192	12,238
**Transcript mean size cDNA (bp)**	2,105.83	1,894.44
**Transcript median size cDNA (bp)**	1,889	1,671
**Min cDNA**	402	200
**Max cDNA**	11,557	16,724
**Total exons**	271,900	655,747
**Exons per transcript**	6.23	5.52
**Exon mean size (bp)**	337.81	343.29
**CDS mean size (bp)**	257.15	242.85
**Transcript mean size CDS (bp)**	1,435.87	1,135.21
**Transcript median size CDS (bp)**	1,275	948
**Min CDS**	0	0
**Max CDS**	10,899	16,092
**Intron mean size (bp)**	469.06	505.98
**5'UTR mean size (bp)**	230.09	270.55
**3'UTR mean size (bp)**	373.81	419.66

Table S7. List of Species used for cross species protein alignment.

**NCBI_RefSeq_ID**	**Species**
GCF_000003195.3	*Sorghum bicolor*
GCF_000005505.3	*Brachypodium distachyon*
GCF_000263155.2	*Setaria italica*
GCF_001433935.1	*Oryza sativa*
GCF_002162155.2	*Triticum dicoccoides*
GCF_002211085.1	*Panicum hallii*
GCF_002575655.2	*Aegilops tauschii subsp. strangulata*
GCF_016808335.1	*Panicum virgatum*
GCF_902167145.1	*Zea mays*
GCF_904849725.1	*Hordeum vulgare*

Table S8. BUSCO evaluation results of (a) genome assembly and (b) gene model prediction

	**(a) Genome assembly**		**(b) Gene model prediction**	
	Value	%	Value	%
**Complete BUSCOs**	4852	99.1	4893	99.9
**Complete and single-copy BUSCOs**	430	8.8	153	3.1
**Complete and duplicated BUSCOs**	4422	90.3	4740	96.8
**Fragmented BUSCOs**	3	0.1	1	0.0
**Missing BUSCOs**	41	0.8	2	0.1
**Total BUSCO groups searched**	4896		4896	
